# Interactive effects of nitrogen addition, warming and invasion across organizational levels in an old-field plant community

**DOI:** 10.1093/aobpla/plu061

**Published:** 2014-10-08

**Authors:** Elise S. Gornish

**Affiliations:** 1 Department of Biological Sciences, Florida State University, Tallahassee, FL 32304, USA; 2 Present address: Plant Sciences, University at California, Davis 95616, USA

**Keywords:** *Ambrosia artemisiifolia*, climate change, leaf toughness, *Pityopsis aspera*, richness, RWC, scale effects.

## Abstract

Response to global change is dependent on the level of biological organization (e.g. the ecologically relevant spatial scale) in which species are embedded. For example, individual responses can affect population-level responses, which, in turn, can affect community-level responses. Although relationships are known to exist among responses to global change across levels of biological organization, formal investigations of these relationships are still uncommon. I conducted an exploratory analysis to identify how nitrogen addition and warming by open top chambers might affect plants across spatial scales by estimating treatment effect size at the leaf level, the plant level and the community level. Moreover, I investigated if the presence of *Pityopsis aspera*, an experimentally introduced plant species, modified the relationship between spatial scale and effect size across treatments. I found that, overall, the spatial scale significantly contributes to differences in effect size, supporting previous work which suggests that mechanisms driving biotic response to global change are scale dependent. Interestingly, the relationship between spatial scale and effect size in both the absence and presence of experimental invasion is very similar for nitrogen addition and warming treatments. The presence of invasion, however, did not affect the relationship between spatial scale and effect size, suggesting that in this system, invasion may not exacerbate or attenuate climate change effects. This exercise highlights the value of moving beyond integration and scaling to the practice of directly testing for scale effects within single experiments.

## Abstract (precis)

Although relationships are known to exist among responses to global change across levels of biological organization, formal investigations of these relationships are still uncommon. I conducted an exploratory analysis to identify how effect size of nitrogen addition, warming, and invasion might affect plants across spatial scales. I found that, overall, spatial scale significantly contributes to differences in effect size. The presence of invasion, however, did not affect the relationship between spatial scale and effect size. This exercise highlights the value of moving beyond integration and scaling to the practice of directly testing for scale effects within single experiments.

## Introduction

Organisms respond to global environmental changes in many ways, including modifications in phenology (e.g. Edwards and Richardson 2004; Moller 2008), decreases in species richness (e.g. Hansen *et al.* 2001) and species abundance (e.g. Gilbert *et al.* 2008), and rapid evolution (Parmesan 2006). Underlying these broad, population and community-level responses are individual demographic traits, which also respond to environmental changes in complex ways (Jongejans *et al.* 2010; Hoving *et al.* 2013). For example, using a meta-analysis, Chalcraft *et al.* (2008) showed that larger-scale, across-site responses to nitrogen enrichment were contingent on the smaller scale primary productivity within sites. Top-down effects have also been documented (e.g. Ludwig *et al.* 2000), as have complex multidirectional effects across spatial scales (e.g. Browning *et al.* 2012). Collectively, these studies suggest that research attempting to identify the more comprehensive implications of climate change requires experiments that can explicitly capture effects across spatial scales which are organized by ecologically relevant biological hierarchies (i.e. from individual plant organs, such as a single leaf, to vegetation canopies) (Ozinga *et al.* 2013).

**Figure 1.**
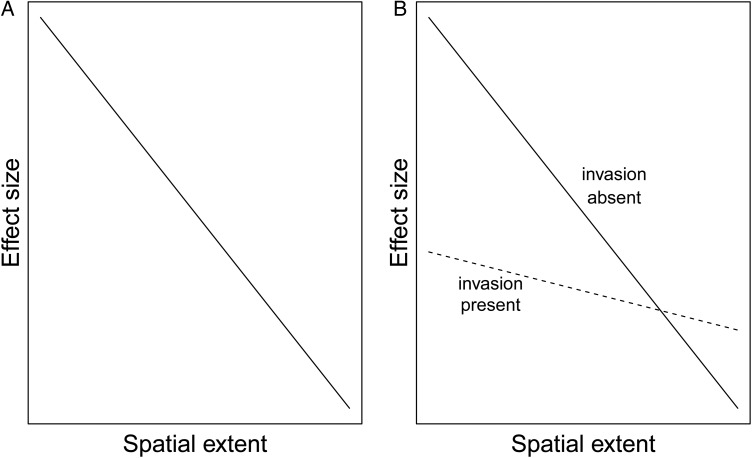
Expectations for the effect of global change treatments (A) and the interaction of global change treatments and invasion (B) on effect size of responses across spatial extents.

A relatively recent review found evidence for a dampening effect at increasing spatiotemporal scales in studies of biotic response to global change (Leuzinger *et al.* 2011). Specifically, they found that effect size (% deviation from control treatments) shows a negative relationship with the (i) number of treatment factors used in an experiment, (ii) temporal extent of an experiment and (iii) spatial extent of an experiment (Fig. 1A). Effect size is expected to decrease as experimental duration increases, partly due to the widely documented phenomenon of acclimation by the experimental species to the particular treatment simulating global change (e.g. Pedrol *et al.* 2000; Maherali *et al.* 2002; Rogers and Ellsworth 2002; Wu *et al.* 2012). Alternatively, an increase in treatment complexity and spatial extent of an experiment can increase the number of factors modifying a response to simulated or natural global change. These additional factors render cause–effect relationships less immediate. This may largely be due to attenuation of effect sizes through antagonistic responses (i.e. Levin 1993; Dieleman *et al.* 2012). For example, although several factors might be involved in driving a response of a leaf to an experimental treatment (e.g. herbivore presence, light availability, etc.), the effect size of a leaf-level response such as leaf N content is modified primarily by chemical processes occurring inside of a single leaf or stem (e.g. Reid *et al.* 1998). As higher spatiotemporal levels are considered, the number of factors that play a role in modifying the effect size of a response must increase. This is because each level of organization will include at least the factors driving the response at lower levels (e.g. Chesson *et al.* 2005), in addition to those factors only present at higher levels. For example, the factors that modify effect size of a tree-level response include leaf-level phytochemicals and herbivores, as well as soil properties, plant–plant and plant–atmosphere interactions (e.g. Saxe *et al.* 1998). In contrast, factors that modify effect size of a leaf-level response only include those relevant at the leaf level, namely the first two (photochemicals and herbivores). Since an increase in the number and diversity of factors in a system is generally considered to increase ecological complexity (e.g. Parrot 2010), this could lead to a dilution of effect size with increasing spatial perspective, as described above.

Here, I describe a single experiment in which the effects of two factors associated with global change (nitrate addition and elevated temperature) are assessed at different levels of spatial organization: at the leaf level, at the plant level and at the community level. This experiment allowed me to explore the general relationship between spatial scale and vegetation response to global change treatments. Moreover, an additional treatment, simulated invasion through the introduction of a previously absent plant species into experimental plots, allowed me to assess if increasing ecological complexity serves to extend the distance (as defined above) between treatment and response, thereby dampening the effect size of a global change treatment.

I expected to find a negative relationship between effect size and the spatial scale at which the treatment response was assessed (Leuzinger *et al.* 2011; Fig. 1A). The interaction of experimental invasion with warming and elevated nitrogen, however, was expected to have a less straightforward effect. First, because invasion can cause direct and indirect effects (thereby increasing ecological complexity) across all levels of biological organization (White *et al.* 2006), the presence of the invasion treatment was expected to reduce effect size across all spatial extents. Second, I expected the slope of the relationship between spatial extent and effect size to become less steep in the presence of invasion. A meta-analysis by Vila *et al.* (2011) suggests that the effects of invaders are larger at lower levels of ecological organization compared with those at higher levels of ecological organization. A larger absolute effect of invasion at these lower levels suggests a bigger disparity in effect size at the leaf and plant level compared with the community and ecosystem level (Fig. 1B).

## Methods

### Experiment

This study was conducted between 2011 and 2012 in a 1.6-hectare-old field at Tall Timbers Research Station (30°39′06.37″N, 84°14′58.30″W), just south of the Florida–Georgia border (last used for agriculture ca. 150 years ago). There is a diverse native plant community in the field, dominated by grasses and legumes, and it is surrounded on all sides by a mixed loblolly shortleaf pine forest. The field was disked annually, and the soil type is a slightly acidic sandy loam (pH ranges from 5.2 to 6.0). Precipitation at the site averages 100 cm per year, and the average annual air temperature is 20 °C.

The experiment was nested within a larger design and is a randomized complete block split-plot design with three main factors: nitrogen addition, warming and experimental invasion, for a total of eight treatment combinations. To minimize leaching of nitrogen between sub-plots, the plots were arranged in a split-plot design, with nitrogen treatments applied to blocks comprising eight plots. Each block of treatments was replicated five times, for a total of 40 plots. Each plot was 4 m^2^, but measurements were only collected from the center 1 m^2^ as a precaution against edge effects. Plots were separated by 1 m, and rows between plots were mowed annually.

#### Nitrogen

Six applications of equal amounts of sodium nitrate (NaNO_3_) were applied during the growing season (April–September) in 2011 and 2012, 5 cm below the soil surface of treatment plots to give a total amount of 4 N g m^−2^ per year. This amount was based on projected dry + wet nitrogen deposition rates for northern Florida (Holland *et al.* 2005), and exists on the more extreme edge of expected increases in deposition (NADP 2010). Each application was followed by the application of 800 mL of water to flush the nitrogen below the soil surface. The nitrogen treatment significantly increased foliar nitrogen of experimental plants (see Gornish 2014). Plots not receiving nitrogen received comparable amounts of water.

#### Warming

Warming was applied to experimental plots by erecting open-top hexagonal chambers constructed of a wooden frame (2.54 × 5 cm boards of pressure treated YellaWood^®^) wrapped with 4 mm clear polyethylene plastic sheeting (Marion *et al.* 1997) in August 2011. The base of the chamber was 2.4 × 2 m and the top of the chamber was 1.7 × 0.8 m. Each panel was 0.6 m tall. Due to uneven microtopography, the chambers sat ∼3 cm off the ground, allowing for air circulation beneath the base of the greenhouses (Havstrom *et al.* 1993) and the unimpeded movement of ground dwelling insects into and out of the warmed plots. The chambers increased the average ambient temperature by 2.5 °C (Gornish 2014), and on average, chambers increased night temperatures 25 % more than they increased day temperatures. The chambers were left in the field for the full year of the experiment.

#### Invasion treatment

Invasion was simulated by experimentally introducing adult (>1-year old) individuals of the perennial composite *Pityopsis aspera* Shuttlw. Ex Small (Asteraceae) into experimental plots in August 2011. The goldenaster, commonly known as pineland silkgrass, is an herbaceous dicot common in xeric sandhill habitats (Myers and Ewel 1990) in northern Florida and south Georgia. It is self-incompatible (Bowers 1972), reproducing both vegetatively and sexually. *Pityopsis aspera* was used as an experimental invader because it typically occurs in the understorey of surrounding forests and, therefore, could be reasonably expected to colonize the old field through the range filling as a response to a changing climate. The experimental old field is within the range of *P. aspera*, which occurs in north Florida, but is devoid of *P. aspera* individuals. *Pityopsis aspera* individuals were planted at a density of 20 per plot (10 individuals in the center 1 m^2^ of the plot and 10 in the periphery of the 4 m^2^ plot). Twenty holes were excavated and refilled in all plots that did not receive transplants, to simulate disturbance due to transplanting.

### Measurements

Responses to the experimental treatments were assigned to the spatial level at which they are mostly relevant. All leaf- and plant-level measurements were taken from *Ambrosia artemisiifolia* L. (annual ragweed), an abundant native annual composite that was naturally found in all of the experimental plots. This cosmopolitan species emerges in late spring, can grow to a substantial height (∼1 m) and produces copious windborne pollen, contributing to its weedy status outside of the USA (Gladieux *et al.* 2011). This species has been shown to respond favorably to nitrogen addition (e.g. Leskovsek *et al.* 2012) and warming (e.g. Essl *et al.* 2009).

Response variables were organized from small to large based on three predictions. First, I used common hierarchical organizational approaches where larger scale factors are composed of a collection of smaller scale factors (e.g. Baldocchi 1993; Dent *et al.* 2001). Second, I assumed that larger scale factors would be involved in more intraspecific and interspecific interactions (Chesson 1998). Third, I assumed that changes in larger scale factors would take more time than changes in smaller scale factors (e.g. Woodmansee 1988).

At the leaf level, I measured relative water content (RWC) and leaf toughness were measured. Foliar RWC can be related to both water availability and stomatal function (Mann *et al.* 2011), both of which can be modified directly and indirectly by factors associated with climate change. Relative water content was measured using rapid estimate procedures modified from Smart and Bingham (1974). In June 2012, three leaves were collected at random from two randomly chosen *A. artemisiifolia* individuals in each plot. The leaves were wrapped in plastic wrap and placed in a dark container until weighing. Samples were first weighed to determine fresh weight (FW), and were then reweighed to determine turgid weight (TW) after being immersed in deionized water for 3 h in a dark fridge. Finally, the samples were blotted to dryness and placed in an oven at 85 °C for 24 h and then reweighed for dry weight (DW):

$$\mathrm{RWC}=\frac{\mathrm{FW}-\mathrm{DW}}{\mathrm{TW}-\mathrm{DW}}$$

Relative water content values for leaves in each plot were averaged for a single plot RWC value.

I also measured leaf toughness in June 2012 to assess treatment effects at the lowest spatial scale. Leaf toughness can be related to plant defence against biotic and abiotic stresses (Read and Stokes 2006; Dominy *et al.* 2008) and can play a role in driving plant tolerance to changing environmental factors (Poorter 2009). In June 2012, I collected the top two leaves from two randomly chosen *A. artemisiifolia* individuals in each plot. Leaf toughness was calculated by measuring the weight of sand necessary to puncture a hole through the center of a single leaf (Feeny 1970). Leaf toughness values were averaged among the four leaves collected per plot.

For plant-level response, I measured plant height, which is strongly correlated with the above-ground plant biomass and other important traits (Falster and Westoby 2003), and is an important component of response to environmental variation. In June 2012, the height of the three largest (generally not yet flowering) individuals of *A. artemisiifolia* was measured to the nearest centimetre in each plot. For community-level response, I measured species diversity and functional diversity of the plant community visually in each plot in August 2012, when most species are at peak biomass. Species diversity was quantified by visually counting the unique number of plant species in each plot. Functional groups were chosen to match the types of plant groups that drive succession in abandoned fields. For example, old fields are generally dominated by graminoids, legumes and annual herbs. As succession progresses, perennial herbs, vines and woody species tend to be dominant (Hermy and Verheyen 2007). Plants were therefore divided into functional groups based on a combination of lifespan, nitrogen-fixing capability, amount of woody materials and growth form. Functional groups included in this analysis were perennial and annual herbs, legumes, graminoids, woody plants and vines.

Due to the breadth of response variables included in this study, variation in measurement precision was likely not similar across the data set. Measurement error was expected to be higher in leaf and plant variables compared with numerical community variables, and these errors could have propagated into effect size estimation (Garrod *et al.* 2013; see below). Despite these limitations, the data presented are still useful for exploring concepts related to the role of spatial scale in modifying response to factors associated with global change.

### Analysis

Using MetaWin (Rosenberg *et al.* 1999), I used the log response ratio (ln *R*) as my estimate of effect size for all measured responses, calculated as

$$\ln\;R=\ln\left(\frac{X^{E}}{X^{C}}\right)$$

where *X*^E^ and *X*^C^ are means of the experimental and control groups, respectively. I used the log response ratio as this metric can reduce the effect of plant size across scales on our response variables (Hedged *et al.* 1999).

I was interested in exploring if spatial scale and the presence of invasion contributed to differences in response variables; however, due to small sample sizes (replicates were ‘taken up’ by calculating effect sizes), this analysis on the effect sizes themselves was descriptive. Additionally, I used analysis of variance (ANOVA) to identify the main and interactive effects of invasion (absence and presence) and spatial scale (leaf, plant and community) on response variables (*n* = 5 for each response) overall, as well as for each of the main treatments (nitrogen addition and warming). Patterns detected in this analysis could suggest dynamics describing the relationship between effect size and the spatial scale of observation and perhaps encourage further investigations. All analyses were conducted in R (version 2.15.1, R Development Core Team 2012).

## Results

Relative water content was mostly unaffected by the treatments (Table 1), although the nitrogen + warming interaction reduced RWC in the leaves of *Ambrosia artemisiifolia* relative to the control plots. Warming and nitrogen as main effects increased leaf toughness in the absence of invasion, but the pattern was reversed in the presence of invasion (Table 1). Height of *A. artemisiifolia* was maintained or reduced in response to all treatment main effects, but was slightly increased in the presence of the nitrogen + warming + invasion treatment. At the community level, both functional and species richness were relatively low across all plots and, unexpectedly, were generally unaffected by all experimental treatments (Table 1). Analysis of variance results suggest that, overall, the effect of global change treatments changed with spatial scale (*F*_2, 24_ = 7.67, *P* = 0.003). The interaction between experimental invasion and spatial scale also contributed to differences in effect size overall (*F*_2, 24_ = 4.71, *P* = 0.02).

**Table 1.** Means and (SD) of all responses, across treatments.

	RWC (%)	Leaf toughness (g)	Height (cm)	Functional richness (#)	Species richness (#)
Control					
Invasion absent	47.0 (8.5)	80.6 (9.8)	99.3 (11.5)	4.8 (1.0)	9.4 (2.9)
Invasion present	47.0 (7.7)	79.9 (10.3)	103.5 (14.9)	4.8 (0.7)	9.1 (2.9)
Nitrogen					
Invasion absent	50.0 (4.9)	90.6 (30.3)	99.8 (15.5)	5.1 (0.9)	10.8 (4.1)
Invasion present	47.0 (4.9)	66.8 (10.6)	96.5 (18.6)	5.5 (0.5)	11.1 (2.8)
Warming					
Invasion absent	46.0 (9.1)	86.6 (12.6)	97.6 (10.1)	5.4 (0.5)	10.3 (2.3)
Invasion present	46.0 (6.9)	68.5 (19.7)	102.8 (12.6)	5.0 (0.8)	12.2 (2.1)
Nitrogen + warming					
Invasion absent	42.0 (7.6)	76.8 (24.9)	102.4 (13.2)	5.3 (0.7)	10.5 (2.8)
Invasion present	44.0 (6.8)	73.2 (6.6)	99.1 (15.2)	5.1 (0.7)	10.3 (2.4)

### Nitrogen

**Figure 2.**
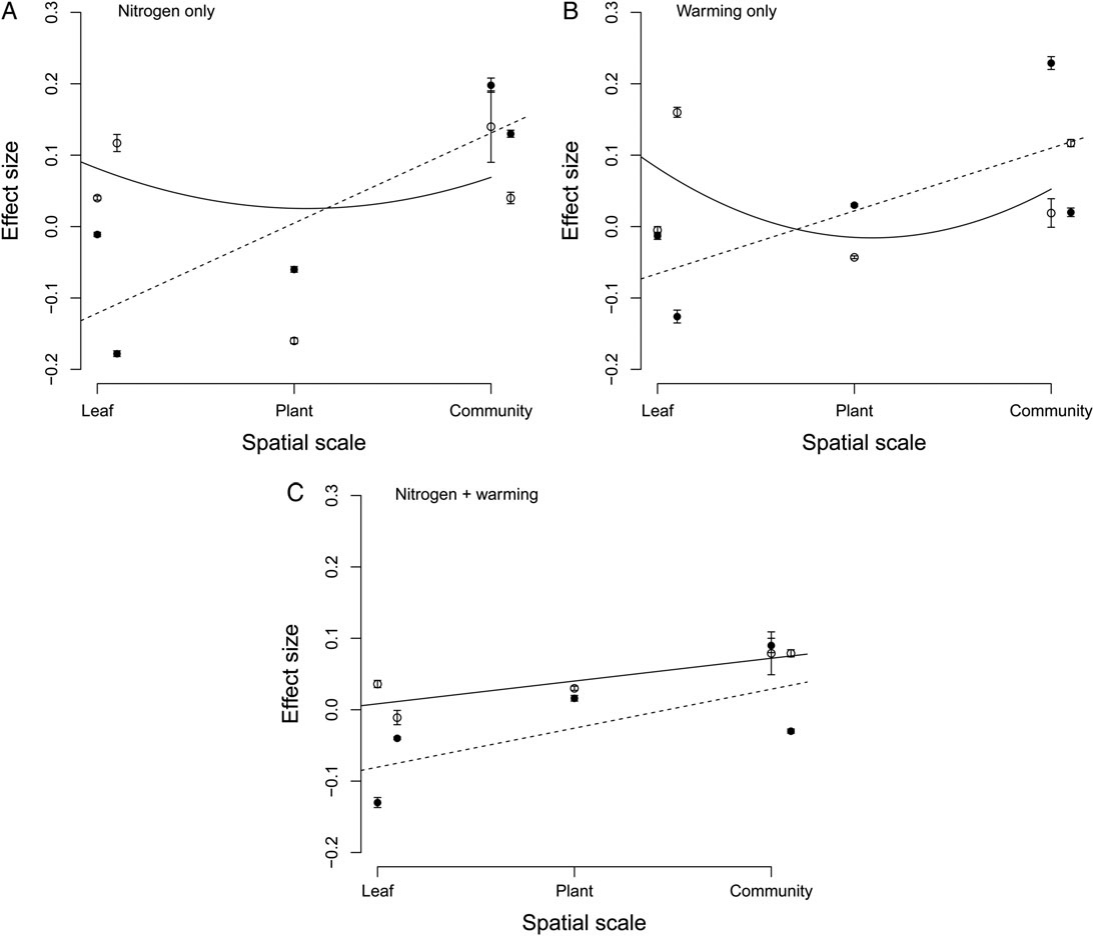
Effect sizes and effect size variance for global change treatments in the absence (empty points, solid line) and presence (filled points, dotted line) of the invasion treatment: (A) nitrogen only, (B) warming only and (C) nitrogen and warming. Loess splines are included to highlight relationships. The order of response variables across the *x*-axis is: RWC and leaf toughness (leaf level); height (plant level); species richness and functional richness (community level).

Variance associated with effect size was larger in the absence of invasion (Fig. 2A). Spatial scale contributed to differences in effect size in the presence of nitrogen (*F*_2,12_ = 6.02, *P* = 0.01). In the presence of invasion, there appeared to be a positive relationship between spatial scale and effect size of nitrogen addition. However, there was no main effect of invasion on effect size (*F*_1,12_ = 3.48, *P* = 0.08), and no interactive effect of spatial scale and invasion (*F*_2,12_ = 2.52, *P* = 0.11).

### Warming

Patterns of effect size across spatial scales in the presence and absence of invasion and warming were almost identical to those identified for the nitrogen treatment (Fig. 2B). Analysis of variance results for plots exposed to warming showed that there was also no significant main effect of spatial scale (*F*_2,12_ = 3.05, *P* = 0.07) or invasion (*F*_1,12_ = 1.65, *P* = 0.21) on effect size. There was also no significant interaction between the two factors (*F*_2,12_ = 2.05, *P* = 0.16).

### Nitrogen + warming

The nitrogen + warming effect sizes displayed the most similar effect sizes across spatial scales. The shallow, positive relationships between effect size and spatial scale in the absence and presence of invasion were not significant (Fig. 2C).

## Discussion

Although scale effects are common (Wiens 1989) and play an important role in driving ecological dynamics (e.g. Villellas *et al.* 2013), experiments that attempt to directly assess the relationship between the ecological response to changing environmental factors and spatial scale are uncommon. Understanding the role of spatial scale in driving ecological dynamics is necessary for developing a conceptual framework in which to consider biological response to a changing environment (e.g. Ibanez *et al.* 2014). Although I am aware that the interpretation of the data depends on how the treatment responses are defined on the spatial scale, my experimental approach facilitated an exploration of how the spatial scale of response can contribute to different effect sizes of nitrogen addition and warming. Also, over time, response patterns may change, but including the temporal component was beyond the scope of this study. In the following, I concentrate on the effect of spatial scales on plant responses. Further, I look at the role of invasion in modifying scale effects and how effect sizes are impacted by single versus combined treatment effects.

In the presence of invasion overall, I found a trend of increasing effect size with increasing spatial scale. Although these results correspond with observations recorded in other studies (Strengbom *et al.* 2006; Chalcraft *et al.* 2008; Oba *et al.* 2008), they do not support initial hypotheses (Fig. 1). A possible explanation is that my original hypotheses were partly predicated on the assumption that response rates at small scales are faster than those occurring at larger scales (Heffernan *et al.* 2014). A larger effect might then be expected at smaller spatial scales for short-term experiments (like the one described in this paper). However, it is possible that a single year of exposure to experimental treatment was not adequate time for responses at all spatial scales to occur. Moreover, if smaller scale responses occurred immediately after treatment application, then acclimation could have occurred at these smaller scales by the time data collection occurred, dampening the presumed effect of treatments.

A seeming absence of a contribution from spatial scale or the presence of an invader on responses from plants exposed to the nitrogen + warming treatment was also surprising. The interaction between temperature and nitrogen deposition has been shown to significantly affect plants and plant communities (e.g. Jones and Power 2011). Increasing the number of treatments simulates increasing environmental heterogeneity, subsequently affecting resilience across a system through portfolio effects (Schindler *et al.* 2010). It is possible that increased resilience reduced the magnitude of response across spatial scales, diluting the effect size–spatial scale relationships. However, the trend of lower effect sizes in the combined treatment plots versus the single treatment plots could be confirmed by this study: generally, effect sizes were larger under warming and nitrogen alone than under its combination.

Interestingly, I found that invasion played a role in modifying the relationship between spatial scale and effect size overall. I expected that as the number of relevant processes contributing to an ultimate response across spatial scales increases, the ecological ‘distance’ between cause and effect would expand, subsequently modifying the relationship between effect size and spatial scale. My observation could be due to emergent effects (Didham *et al.* 2007), which are often responsible for invaders having a larger effect on native plants in the presence of resource addition (e.g. Green and Galatowitsch 2001).

## Conclusions

Studies that explicitly explore scale effects are of primary importance to understanding the underlying ecological processes driving large-scale responses. However, most studies that include spatial scale do so indirectly (e.g. Takagi and Miyashita 2014). Results of this study, although exploratory, do suggest that spatial scales play a role in modifying effect sizes of climate change response in plants. Although I found signals of scale effects in response to experimental treatments overall, these signals can be context dependent (Dent *et al.* 2001), and perhaps a different type of treatment (elevated CO_2_, for example) may elicit different relationships. Clearly, it is difficult to draw robust conclusions from a single case study, as only a small number of species and treatment effects are involved. The detection of overarching scaling effects often requires a large number of studies in order to obtain a reasonable signal-to-noise ratio. Nevertheless, I argue that it is important to use single case studies to verify the effects of spatial (and temporal) scaling. Such efforts have become more common recently (Heffernan *et al.* 2014), and it must become a more regular part of experimental research in order to develop our understanding of the complex relationships driving ecological patterns.

## Sources of Funding

Funding was provided by Tall Timbers Research Station, T. E. Miller and Florida State University.

## Contributions by the Authors

E.S.G. executed the experiment, collected and analysed all data and wrote the manuscript.

## Conflicts of Interest Statement

None declared.
